# In vitro metabolism of beclomethasone dipropionate, budesonide, ciclesonide, and fluticasone propionate in human lung precision-cut tissue slices

**DOI:** 10.1186/1465-9921-8-65

**Published:** 2007-09-20

**Authors:** Ruediger Nave, Robyn Fisher, Nigel McCracken

**Affiliations:** 1 DMPK, Nycomed GmbH, Konstanz, Germany; 2 Vitron, Inc., Tucson, Arizona, USA

## Abstract

**Background:**

The therapeutic effect of inhaled corticosteroids (ICS) may be affected by the metabolism of the drug in the target organ. We investigated the *in vitro *metabolism of beclomethasone dipropionate (BDP), budesonide (BUD), ciclesonide (CIC), and fluticasone propionate (FP) in human lung precision-cut tissue slices. CIC, a new generation ICS, is hydrolyzed by esterases in the upper and lower airways to its pharmacologically active metabolite desisobutyryl-ciclesonide (des-CIC).

**Methods:**

Lung tissue slices were incubated with BDP, BUD, CIC, and FP (initial target concentration of 25 μM) for 2, 6, and 24 h. Cellular viability was assessed using adenosine 5'-triphosphate content and protein synthesis in lung slices. Metabolites and remaining parent compounds in the tissue samples were analyzed by HPLC with UV detection.

**Results:**

BDP was hydrolyzed to the pharmacologically active metabolite beclomethasone-17-monopropionate (BMP) and, predominantly, to inactive beclomethasone (BOH). CIC was hydrolyzed initially to des-CIC with a slower rate compared to BDP. A distinctly smaller amount (approximately 10-fold less) of fatty acid esters were formed by BMP (and/or BOH) than by BUD or des-CIC. The highest relative amounts of fatty acid esters were detected for BUD. For FP, no metabolites were detected at any time point. The amount of drug-related material in lung tissue (based on initial concentrations) at 24 h was highest for CIC, followed by BUD and FP; the smallest amount was detected for BDP.

**Conclusion:**

The *in vitro *metabolic pathways of the tested ICS in human lung tissue were differing. While FP was metabolically stable, the majority of BDP was converted to inactive polar metabolites. The formation of fatty acid conjugates was confirmed for BMP (and/or BOH), BUD, and des-CIC.

## Background

Inhaled corticosteroids (ICS) are the standard first-line anti-inflammatory therapy for the management of persistent asthma [1]. ICS increase pulmonary function and decrease airway hyperresponsiveness, symptom severity, acute exacerbations [2], hospitalizations, and asthma mortality rates [3]. Binding of an ICS to the glucocorticoid receptor in the lung leads to a pleiotropic reduction in the expression of multiple pro-inflammatory pathways by reversing histone acetylation of the activated genes [4,5].

The preferred way to administer a corticosteroid in asthma therapy is by inhalation. This route delivers drug straight to the lung for local activity and minimizes the systemic side effects associated with oral administration. Nevertheless, even treatment with ICS may cause both local and systemic side effects. Deposition of the drug in the oropharynx may cause local side effects, including oropharyngeal candidiasis, dysphonia, reflex cough, bronchospasm, and pharyngitis [6,7]. Direct absorption of the active drug into the systemic circulation across the mucosa of the oropharynx and airways or absorption of swallowed drug via the gastrointestinal tract may result in systemic side effects. Skin thinning and bruising, cataracts, growth retardation, accelerated bone loss, and hypothalamic-pituitary-adrenal axis suppression have been observed in patients receiving long-term, high-dose ICS treatment [8,9].

Pharmacokinetic (PK) properties of ICS are related to safety and efficacy of the drug. The efficacy of an ICS is dependent on high glucocorticoid receptor binding, high pulmonary deposition and retention, enhanced lipophilicity, and lipid conjugation. Safety is optimized by low oral bioavailability, high protein binding, and rapid systemic clearance [10,11]. Beclomethasone dipropionate (BDP), budesonide (BUD), and fluticasone propionate (FP) are widely used ICS and have been on the market in Asia, America, and Europe for several years. Ciclesonide (CIC) is a novel, nonhalogenated ICS that has been developed to incorporate PK properties that lead to high efficacy and an improved safety profile [11-15]. The prodrug CIC is administered as an inactive parent compound to the lower airways, where it is converted to its pharmacologically active metabolite desisobutyryl-ciclesonide (des-CIC) by endogenous esterases [12,16-19] Des-CIC has a high affinity to the glucocorticoid receptor, comparable with beclomethasone-17-monopropionate (BMP), whereas BUD has a slightly lower and FP a higher binding affinity [11]. Within the lung cells, des-CIC and BUD undergo reversible esterification with fatty acids at the C-21 position of the molecules. The formed fatty acid conjugates may serve as a depot that slowly releases des-CIC and BUD in the lung [16,20-23] Hydrolysis of BDP at positions 17 and 21 results in three different metabolites, ie BMP, beclomethasone-21-monopropionate (21-BMP), and beclomethasone (BOH). BMP is the active metabolite, whereas BOH and 21-BMP have a very low binding affinity to the glucocorticoid receptor [11,24].

In the present study we investigated the *in vitro *metabolism of BDP, BUD, CIC, and FP in the target organ lung, using human lung precision-cut tissue slices, to evaluate the major factors influencing the retention time of these ICS in the lung.

## Methods

### Materials

BUD, CIC, des-CIC, des-CIC-palmitate, des-CIC-oleate, and FP were synthesized by ALTANA Pharma AG, Konstanz, Germany. BDP and BMP were obtained from MDS Pharmaservices (Fehraltorf, Switzerland).

Waymouth's MB752/1 powdered medium without phenol red was purchased from Gibco Laboratories (Grand Island, NY, USA). Fetal bovine serum and Fungi-Bact solution were purchased from Irvine Scientific (Santa Ana, CA, USA). Electrophoresis-grade agarose was obtained from ICN Biochemicals (Cleveland, OH, USA), and tracheal balloon catheters were obtained from Mallinchrodt Critical Care (Glen Falls, NY, USA). The dynamic roller culture incubators, Brendel/Vitron tissue slicer, titanium roller inserts, cordless coring tools, and V-7 cold preservation solution were manufactured by Vitron, Inc. (Tucson, AZ, USA). Water was Millipore filtered. All other reagents were obtained from Merck (Darmstadt, Germany) and were of professional analysis quality except methanol and ethanol, which were HPLC gradient grade.

### Human tissue collection

The American Association of Human Tissue Users supplied three lungs. The lungs were accepted by The American Association of Human Tissue Users from American procurement agencies that followed donor consent procedures according to accepted medical and ethical standards as outlined by the Uniform Anatomical Gift Act. Tissues were negative for infectious agents including HIV and hepatitis. Organs were obtained under transplantation, but were not viable for transplantation because of anatomic irregularities, histological findings, age of donor, time in transit, or health status of the recipient.

### Preparation of tissue slices

Lung slices were prepared at Vitron, Inc. (Tucson, AZ, USA) as previously described [25]. In brief, the lungs were inflated with 2 to 4 L of 0.5% agar/Waymouth's medium mixture at 37°C and were stored at 4°C for 30 to 60 min. Each lung was cut into 2 cm thick slabs, placed on dental wax, and tissue cylinders of 8 mm diameter were prepared using a cordless coring tool. 400 μm thick slices were cut from the cylinders using a Brendel-Vitron tissue slicer [26].

### Tissue incubation

Three lung slices from each donor were floated onto titanium roller inserts, blotted, and loaded horizontally into glass scintillation vials containing 1.7 mL of Waymouth's MB752/1 tissue culture medium fortified with 10% fetal calf serum, 0.35 g/L L-glutamine, 10 mL/L Fungi-Bact, and 84 μg/mL gentamicin sulfate.

Three vials were used per substrate and time point. A stock solution of the substrate was added prior to dispensing the media into vials. The final substrate concentration was 25 μM. The vials were closed with caps that had central 2-mm holes, placed in a Dynamic Organ Culture incubator (Vitron, Inc., Tucson, AZ, USA) at 37°C, and exposed to 95% O_2 _and 5% CO_2_. The human tissue slices were incubated for 2, 6, and 24 h, and cellular viability of human lung slices was assessed using separate incubation samples (see assessment of cellular viability). Slices and media from each vial were harvested separately into separate tubes and stored at -80°C. Metabolism samples containing either tissue or media were shipped on dry ice from Vitron, Inc. to ALTANA Pharma AG for analysis.

### Assessment of cellular viability

Cellular viability was determined by measuring adenosine 5'-triphosphate (ATP) content and protein synthesis in human lung tissue. ATP content was measured using a luciferin-luciferase assay [27]. Tissue slices were homogenized in 1 mL 10% trichloroacetic acid and were immediately frozen at -80°C until ATP content was measured using a luminometer. Protein synthesis was measured by the addition of 0.3 μCi [^3^H]-leucine/mL to tissue slice incubations. Tissue slices were sonicated in 1 mL distilled water, and a 20 μL aliquot was used to determine protein content. The remaining homogenate was precipitated with 5% (w/v) perchloric acid. Samples were centrifuged and the resultant pellet was washed 3 times. The final pellet was dissolved in 0.5 mL of 0.5 N sodium hydroxide and neutralized with 125 μL of 2 N hydrochloric acid. Incorporation of the labeled amino acid into acid-precipitable material was measured by counting a 0.5 mL aliquot of the sample in Bio-Safe II scintillation cocktail. Viability of lung tissue slices was expressed per milligram of protein.

### Sample preparation

The volume of medium was measured at the end of the incubation period. Samples of the incubation medium were prepared by mixing 60 μL of medium with 60 μL of solution for precipitation (0.2 M ZnSO_4_/ACN, 1:1). Following centrifugation for 5 min (1,000 × g Model5414, Eppendorf) at room temperature, the supernatant was assayed by high performance liquid chromatography (HPLC). Tissue samples were homogenized in 1 mL of methanol using ultrasonic treatment (Branson Sonifier 250; Branson Ultrasonics Corporation, Danbury, Conn) and were centrifuged for 5 min (1,000 × g Model5414, Eppendorf) at room temperature. After centrifugation, 333 μL of the supernatant was dried under a gentle stream of nitrogen at 45°C and resuspended in 75 μL of methanol before analysis.

### High-performance liquid chromatography analysis

HPLC analysis was performed using a Hewlett-Packard HP1090 liquid chromatograph (Agilent Technologies Deutschland GmbH, Waldbronn, Germany) with an ultraviolet (UV) detector monitoring at 242 nm. Separation was achieved using a Hypersil MOS column (125 × 4 mm, 5 μm) (Thermo Electron Corp., Dreieich, Germany). A water/ethanol gradient system was run from 30 to 90% ethanol in 10 min followed by an isocratic phase (6 min) with 90% ethanol at a flow rate of 0.75 mL/min. 25 μL of the tissue extract or 100 μL of the supernatant of the medium were injected onto the column. This system was suitable to assay all parent compounds used as substrate and the fatty acid conjugated metabolites. BOH, BDP, BMP, BUD, CIC, des-CIC, des-CIC-oleate, and FP were used as reference compounds. All these compounds have a similar UV response at 242 nm.

## Results

### Donor information

Human lung tissues from 3 human donors were used to study metabolism of four ICS. Donor profiles are summarized in Table 1.

**Table 1:** Donor characteristics

**Donor**	**1**	**2**	**3**
**Age**	19	48	43
**Sex**	Male	Male	Female
**Race**	Hispanic	Caucasian	Caucasian
**Cause of death**	Cerebrovascular	Cerebrovascular	Cerebrovascular
**Medical history**	Healthy	Healthy	Healthy
**Medications**	None	None	None

### Cellular viability

Viability of tissue samples was assessed using ATP content and protein synthesis levels in human lung after incubation of tissue samples with ICS for 2, 6, and 24 h. ATP content ranged from 17.0 to 40.7 nmol/mg protein. Protein synthesis displayed a linear mean increase from 1,930 dpm/mg protein after 2 h of incubation to 15,409 dpm/mg protein after 24 h of incubation (Table 2). Fisher and colleagues have reported that ATP content should exceed 10 nmol/mg protein and protein synthesis should be linear over time in viable human lung tissue [26]. Therefore, all lung tissue samples used in this study were highly viable.

**Table 2:** Viability parameters

**Incubation time [h]**	**Lung from donor**	**ATP Content [nmoles ATP/mg protein]**	**Protein Synthesis [dpm/mg protein]**
	1	36.3 – 37.4	2,688 – 2,797
**2**	2	36.5 – 40.2	1,924 – 2,355
	3	21.0 – 26.2	908 – 939

	1	37.7 – 43.4	4,787 – 5,251
**6**	2	42.1 – 49.1	3,806 – 4,595
	3	20.6 – 24.6	2,898 – 3,481

	1	34.7 – 37.2	13,862 – 14,851
**24**	2	33.6 – 40.7	16,155 – 17,313
	3	17.0 – 23.1	14,268 – 16,455

Data from n = 3 tissue slices per lung.

### Analytics

The HPLC system was suitable to assay all parent compounds used as substrate as well as all known major metabolites. However, the inactive 21-BMP was not available as reference compound. It was assumed that the retention time of BMP and 21-BMP is similar and in some cases shoulders in the BMP peaks were observed. Assuming coeluation of BMP and 21-BMP from the HPLC column and assuming a very similar UV response, the BMP peak may represent partly 21-BMP. Therefore, the amount of active BMP may be overestimated.

Furthermore, the lipophilic fatty acid conjugated metabolites as well as the polar BOH were detectable. Matrix peaks did not influence the detection of drug-related peaks. Results of control samples confirmed the stability of the tested compounds during incubation under the used conditions. Furthermore, matrix peaks of tissue slices and medium could be identified using the HPLC system. Interday variability of all analytes was less than 15%.

### Drug-related material in the incubation medium

Substrates were analyzed in medium after an incubation time of 2, 6, and 24 h. BUD and FP were stable, ie no metabolites were detected in medium, whereas the relative amounts of CIC and BDP decreased over time and metabolites were detected in the incubation medium (Table 3). CIC was metabolized to des-CIC, which was the only metabolite of CIC in the medium. At the end of the incubation period (24 h), the parent compound accounted for 37% of CIC-related material in the medium. In contrast, at 24 h of incubation, there was no BDP detectable in the medium, whereas its metabolites accounted for 91.6% (BOH) and 8.4% (BMP) of BDP-related material (Table 3).

**Table 3:** Relative drug amounts [% of total UV areas] in medium

	**Incubation time [h]**	**Parent compound**	**Active compound**	**Inactive metabolite**
***Beclomethasone ***	2	60.9 ± 7.0	35.7 ± 6.4	3.4 ± 0.8
***Dipropionate^1^***	6	19.1 ± 8.3	53.5 ± 4.3	27.3 ± 4.7
	24	-	8.4 ± 5.3	91.6 ± 5.3

***Ciclesonide***	2	94.9 ± 1.3	5.1 ± 1.3	-
	6	83.2 ± 5.5	16.8 ± 5.5	-
	24	37.4 ± 7.8	62.6 ± 7.8	-

Data are presented as mean ± standard deviation (n = 9).

No metabolites of Bud and FP were detected in medium.

^1^The active compound of BDP may be overestimated as inactive 21-BMP may be included.

### Drug-related material in tissue samples

**Figure 1:**
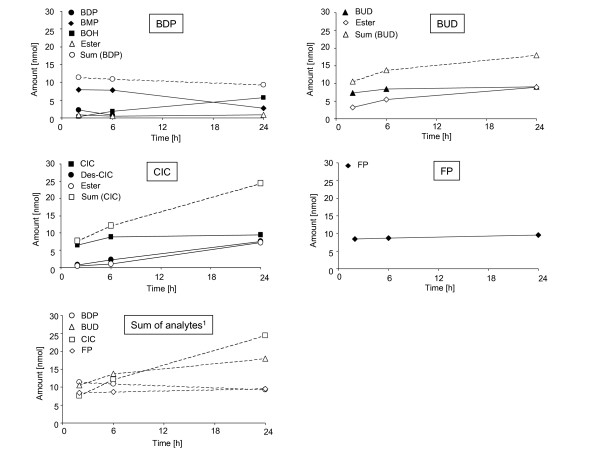
**Mean amounts of parent compounds and metabolites in lung tissue slices over time**. Initial substrate concentration: 25 μM. ^1^Sum of analytes = Pool for active drug detectable in human lung slices after 2, 6, and 24 h of incubation. BDP = beclomethasone dipropionate, BUD = budesonide, CIC = ciclesonide, FP = fluticasone propionate.

In contrast to the medium samples, fatty acid esters were detectable in tissue extracts. Furthermore, metabolic differences between the ICS were obvious in tissue extracts. The relative amounts of BDP, BUD, CIC, FP, and their respective metabolites in human lung tissue at 2, 6, and 24 h of incubation are presented in Table 4. Absolute amounts of the drugs and their metabolites are presented in Figure 1 and Table 5.

**Table 4:** Relative drug amounts [% of total UV areas] in human lung tissue slices

	**Incubation time [h]**	**Parent compound**	**Active compound**	**Fatty acid esters**	**Inactive metabolite**
***Beclomethasone ***	2	21.4 ± 9.8	72.1 ± 9.2	2.9 ± 1.1	3.6 ± 0.8
***dipropionate^2^***	6	9.2 ± 7.9	66.6 ± 17.7	4.4 ± 2.6	19.8 ± 10.0
	24	-	26.6 ± 16.8	8.5 ± 7.8	64.9 ± 14.4

***Budesonide^1^***	2	72.2 ± 10.8		27.8 ± 10.8	-
	6	64.9 ± 12.0		35.1 ± 12.0	-
	24	53.6 ± 11.5		46.4 ± 11.5	-

***Ciclesonide***	2	85.0 ± 3.4	9.9 ± 1.4	5.1 ± 2.1	-
	6	73.5 ± 4.7	17.9 ± 1.5	8.6 ± 4.1	-
	24	40.0 ± 7.6	31.5 ± 2.4	28.5 ± 8.8	-

***Fluticasone^1^***	2	100 ± 0.0		-	-
***propionate***	6	100 ± 0.0		-	-
	24	100 ± 0.0		-	-

Data are presented as mean ± standard deviation (n = 9).

^1^Parent and active compound.

^2^The active compound of BDP may be overestimated as inactive 21-BMP may be included.

**Table 5:** Total amount [nmol/vial] of drug related compounds in human lung tissue slices

	**Incubation time [h]**	**Parent compound**	**Active compound**	**Fatty acid esters**	**Inactive metabolite**
***Beclomethasone ***	2	2.19 ± 0.83	7.88 ± 2.21	0.90 ± 0.46	0.38 ± 0.06
***dipropionate^2^***	6	0.72 ± 0.46	7.78 ± 3.80	0.50 ± 0.40	1.89 ± 0.49
	24	-	2.73 ± 2.22	0.87 ± 0.96	5.72 ± 1.20

***Budesonide^1^***	2	7.32 ± 0.99		3.17 ± 2.00	-
	6	8.41 ± 2.15		5.39 ± 4.03	-
	24	9.04 ± 1.14		8.91 ± 5.15	-

***Ciclesonide***	2	6.46 ± 0.92	0.76 ± 0.2	0.40 ± 0.20	-
	6	8.86 ± 1.68	2.17 ± 0.51	1.10 ± 0.73	-
	24	9.53 ± 1.70	7.61 ± 1.31	7.29 ± 3.79	-

***Fluticasone^1^***	2	8.42 ± 1.26		-	-
***propionate***	6	8.62 ± 2.20		-	-
	24	9.51 ± 2.22		-	-

Data are presented as mean ± standard deviation (n = 9).

^1^Parent and active compound.

^2^The active compound of BDP may be overestimated as inactive 21-BMP may be included.

BDP was hydrolyzed to active BMP and to pharmacologically inactive BOH and 21-BMP. As mentioned above, 21-BMP could not be quantified due to coeluation in the BMP peak and therefore the amount of active BMP may be overestimated. BMP was the major metabolite at 2 h and 6 h, whereas BOH was the major metabolite at 24 h. At 24 h, BMP accounted for 26.6% and BOH for 64.9% of BDP-related material. Fatty acid esters were formed by BMP (and/or BOH), but the amount was distinctly smaller (approximately 10-fold less) when compared with the amount of fatty acid esters found for BUD and des-CIC. The relative amount of BUD decreased over time and similar amounts of BUD and its fatty acid esters were detected in the tissue at 24 h. CIC was initially hydrolyzed to des-CIC, which subsequently was conjugated with fatty acids. Both the relative and the mean amounts of des-CIC and fatty acid conjugates of des-CIC increased over time. For FP, no metabolites were detected in the tissue extract. FP was metabolically stable in this test system. Furthermore, the intracellular amount of FP was similar at all time points during incubation.

Based on these results, active metabolites were formed by BDP and CIC. The enzymatic cleavage of BDP to BMP was faster than the formation of des-CIC from CIC (see 2 h data in Table 4). The active metabolite BMP was further metabolized to BOH, whereas the active metabolite des-CIC and the active compound BUD formed intracellular fatty acid esters. These esters may serve as precursors of the active compounds because the conjugation is a reversible process. Parent compounds, active metabolites, and fatty acid esters form a pool of active drug. At 24 h, the mean absolute values for the active drug pools in the lung tissue slices were 9.31 nmol for BDP, 17.95 nmol for BUD, 24.43 nmol for CIC, and 9.51 nmol for FP. The pool of active drug (including parent compound, active metabolite, and fatty acid esters) was larger for CIC than for the other ICS.

## Discussion

Several pharmacokinetic and pharmacodynamic parameters, including bioavailability, receptor binding-affinity, and elimination half-life contribute to the safety and efficacy profile of an ICS. While developing topical corticosteroids for the treatment of asthma, a delicate balance between safety and efficacy must be considered. In the present study, differences in the *in vitro *metabolism of currently available ICS in human lung tissue slices were evaluated with respect to their local effect on pulmonary residence time.

Results of the present study confirm the hypothesis that the metabolism of BDP, BUD, CIC, and FP in the lung is considerably different. This was shown in a direct comparison using human precision-cut tissue slices. Unlike BUD and FP, which are active in their parent form, both BDP and CIC are activated in the airways by esterases to pharmacologically active BMP or des-CIC, respectively. Results from this study confirm previous findings showing that BDP is hydrolyzed to BOH in the cytosol of human lung cells [24]. However, in the current study the formation of BOH was much faster, as was also the case in a previous study in rat lung precision-cut tissue slices [20]. The majority of BMP was inactivated to BOH and only a small amount of esters was formed. In contrast, a considerable amount of des-CIC fatty acid conjugates were detected. Experimentally it was not clear whether BMP, 21-BMP and/or BOH was conjugated with fatty acids, because the amounts of these metabolites were too low for further characterization by mass spectrometry. Similar to des-CIC, BUD formed intracellular free fatty acid esters. A prerequisite for the formation of fatty acid esters is the presence of a steric hindrance-free hydroxyl group at the C-21 position of the ICS. BMP, BOH, BUD, and des-CIC have a free C-21 hydroxyl group that can be reversibly esterified. BUD is the only parent compound with a free C-21 hydroxyl group and, therefore, esterification can start directly once BUD has been taken up by the cell. This may explain the high portion of fatty acid esters of BUD at early time points. FP, which has no free C-21 hydroxyl group, was metabolically stable in the test system because no metabolites were detectable in the tissue and incubation medium.

In this study, a relatively high initial concentration of ICS was used to investigate metabolism in the lung. The concentration of 25 μM was the same as used in previous studies investigating CIC metabolism in precision-cut tissue slices of human and rat lungs [16,20]. In these studies, the use of ^14^C-ciclesonide allowed the estimation of recovery and mass balance. In the present study, although incubations were carried out with non-radiolabelled CIC, the same methods and conditions were used. The overall metabolism rate of CIC in the current investigation was lower than in the previous study, whereas the viability of the cells was comparable in both studies [16]. At the end of the incubation period, the relative percentage of des-CIC was very similar in both studies (29% vs 32%).

The concentration of corticosteroids in the systemic circulation after inhalation depends on the pharmacokinetic properties of the drug, the formulation, and the device, but is in general much lower than the concentration of ICS used in the current study (25 μM). For example, in healthy volunteers, inhalation of CIC 1280 μg via metered-dose inhaler (MDI) resulted in a C_max _of 5.07 μg/L (9.4 nmol/L, geometric mean) [28] and inhalation of FP 1000 μg via Diskhaler^® ^resulted in a C_max _of 0.38 μg/L (0.76 nmol/L, mean) [29]. However, low serum concentrations of corticosteroids do not necessarily mimic the situation in the lung. After inhalation, concentrations of ICS may be significantly higher in the lung compared with concentrations in serum [6]. This was confirmed in three different studies investigating the concentration of drugs in resected lung tissue samples as well as in serum following single inhalation of ICS. After inhalation of 1600 μg BUD via Nebuhaler^®^, the mean concentration of BUD in lung tissue obtained from resections was 5.5 nmol/kg compared with 0.63 nmol/kg in blood plasma [30]. In a similar study, FP concentrations of more than 20 ng/g central lung tissue were detected in a few patients, while the mean concentration in serum at that time was approximately 0.02 ng/mL [31]. After inhalation of 1 mg BDP via a MDI (Aerobec^®^), the median concentration of BMP in bronchial tissue and serum was 4.4 ng/g tissue and 1.2 ng/mL, respectively [33]. Although quantitative differences cannot be excluded, the metabolic pathways of the tested ICS in the lung should be unaffected by the chosen initial drug concentration.

Lipophilicity and lipid conjugation are two distinct PK parameters that can affect the absorption rate of an ICS across pulmonary membranes and influence retention time in the lung. Slow absorption from the lung or pulmonary retention of the drug by some mechanism may enhance the anti-inflammatory effects of the drug without the unwanted systemic side effects.

The amount of FP detected in the lung tissue remained constant during the investigated period of 2 to 24 h. The relative distribution of FP between the tissue and the incubation medium may depend on the lipophilicity and the glucocorticoid receptor affinity of the compound. Because the concentration of the drug is considered to be high compared with the number of available receptor sites, the main factor for uptake into the cells and intracellular residence seems to be the lipophilicity of the drug (logD of FP at pH 7.4 is 3.7 [20]). However, in the present study no data for the early distribution phase were generated that would allow a correlation between lipophilicity and distribution. The uptake of FP and CIC into human alveolar type II epithelial cells (A549) was investigated separately [32]. In contrast to FP, all other tested drugs were metabolized in the lung tissue. The active metabolite of BDP, BMP, was further converted to fatty acid esters and mainly to inactive polar metabolites. Over time, the concentration of drug-related compounds decreased in the tissue and the formed polar metabolites diffused into the medium (91.6% BOH and 8.4% BMP by 24 h).

The intracellular fatty acid conjugation of BUD and des-CIC dramatically increased the lipophilicity of the drugs. No fatty esters were detectable outside the cells. Des-CIC-oleate (logD 13.0) was about 5-fold more lipophilic than BUD-oleate (logD 12.3) [20]. The absolute drug amounts in the lung slices at the end of the incubation period may be correlated with their lipophilicity. CIC (logD 6.1) and des-CIC fatty acid esters are highly lipophilic [20], which suggests that these compounds would be retained in the lung for a prolonged period of time. Furthermore, both compounds are precursors of des-CIC and may thus serve as a pool of active drug. Although the amount of active compounds measured in the human lung tissue slices were similar for BUD, des-CIC, and FP, the amount of drug-related material detected in the tissue after 24 h of incubation was higher for CIC than for the other tested ICS.

Results from this study confirm previous findings that both CIC and BUD reversibly form conjugates with fatty acids [16,19,21-23]

Unlike BDP and CIC, both BUD and FP are inhaled as active compounds. Therefore, drug exposure in the oropharyngeal region may be much higher for BUD and FP than for the active metabolites of BDP [33] and CIC [34,35]. The activation of BDP in the oropharynx seems to be fast, because similar amounts of BDP and BMP were detected in mouth rinsing solutions directly after inhalation of BDP 1000 μg via MDI [33]. In contrast, the activation of CIC in the oropharynx is very low. Only traces of des-CIC were detected in mouth rinsing solutions directly after inhalation of CIC 800 μg via MDI [34,35]. Higher concentrations of BMP compared to des-CIC in the oropharynx might be explained by differences in the velocity of BMP and des-CIC formation as observed in the present study. Administration of CIC as an inactive parent compound and the low oropharyngeal deposition of des-CIC may explain the low incidence of oropharyngeal side effects observed in clinical studies [36].

## Conclusion

In summary, when developing ICS it is necessary to consider the overall balance between safety and efficacy. In this study we saw clear differences between the investigated ICS regarding the residence time of the drugs within the lung tissue slices, because of their different metabolism, lipophilicity, and ability to form lipid conjugates. Highly lipophilic drugs will likely contribute to a high rate of absorption within the lung. Once in the lung, reversible fatty acid conjugation may prolong the local anti-inflammatory activity by slowly releasing active drug from the fatty acid ester depot. Of the currently available ICS, only CIC and BUD undergo lipid conjugation. This concept supports the once-daily dosing efficacy of these drugs that was observed in clinical studies [37,38].

## Abbreviations

ATP- Adenosine triphosphate.

BDP- Beclomethasone dipropionate.

BMP- Beclomethasone-17-monopropinate.

BOH- Beclomethasone.

BUD- Budesonide.

CIC- Ciclesonide.

FP- Fluticasone propionate.

HPLC- High performance liquid chromatography.

ICS- Inhaled corticosteroid.

UV- Ultra violet.

## Competing interests

RN and NM are employees of Nycomed GmbH (formerly ALTANA Pharma AG), Konstanz, Germany, who sponsored this study. The author RF has received honoraria from ALTANA Pharma AG for conducting part of this study.

## Authors' contributions

RN planned the experiments, carried out the HPLC and data analyses, and drafted the manuscript. RF prepared the lung tissue slices, carried out the tissue incubations and the cell viability tests. NM, as head of department, was involved in developing experimental plans and manuscript revisions. All authors read and approved the final manuscript.
